# Supplementary material for: The evolution of the role of nursing in primary health care using Bourdieu’s concept of habitus. A grounded theory study
Source: PLoS One. 2022 May 17;17(5):e0265378. doi: 10.1371/journal.pone.0265378 (PMC9113590; doi:10.1371/journal.pone.0265378)
Supplement: S1 Table — (DOCX) [file pone.0265378.s001.docx]

S1 Table. Interview script (Spanish).

- Llegada: ¿Cómo llegaste a APS?
- Aprendizaje: a la llegada y a lo largo del tiempo.
- Competencias: ¿qué hacías?
- Diferencias y similitudes con tu experiencia laboral anterior
- Relaciones con otras enfermeras
- Relaciones con los directivos
- Relaciones interprofesionales: ¿cómo era el clima laboral?
- Servicios ofrecidos a la población: ¿cuáles fueron los servicios ofrecidos, qué tipo de actividades se realizaron?
- Momentos importantes / críticos
- Características del trabajo de Enfermería
- Aportaciones de Enfermería: ¿cuáles crees que son?
- Perspectivas futuras
