# Supplementary material for: The evolution of the role of nursing in primary health care using Bourdieu’s concept of habitus. A grounded theory study
Source: PLoS One. 2022 May 17;17(5):e0265378. doi: 10.1371/journal.pone.0265378 (PMC9113590; doi:10.1371/journal.pone.0265378)
Supplement: Attachment — Submitted filename: Response letter. 20 July 2021.docx [file pone.0265378.s002.docx]

Anita Estes

PLOS ONE

July 20, 2021

Dear Anita Estes, 
 
Thank you very much, once again, for the follow-up of our PLOS ONE article entitled "The role of nursing in primary health care using Bourdieu’s concept of habitus. A grounded theory study." 
 
In response to your e-mail of July 12, we are pleased to confirm you the following updated Data Availability Statement you proposed, is acceptable:
 
"All relevant data are within the paper. Supporting Information Files are available on request from the University of Cantabria Archive (<http://repositorio.unican.es/>) for researchers who meet the criteria for access to confidential data. This restriction is due to ethical compliance in order to not compromise study participants' privacy, since the dataset derives from clinical studies involving human participants. Relevant data Requests may be sent to Carmen Chasco Vila, Responsable de División de Medicina y Enfermería. Biblioteca Universitaria. Facultad de Medicina. (Telephone number: + 34 942 201 217; E-mail address: carmen.chasco@unican.es)". 
 
Please let us know if there is still any issue that does not fully comply with your requirements, and we will correct it immediately. As we already told you, we are very much looking forward to the possibility of publishing our work in PLOS ONE.
 
Best regards, 
 
Victor Fradejas Sastre
