# Supplementary material for: The evolution of the role of nursing in primary health care using Bourdieu’s concept of habitus. A grounded theory study
Source: PLoS One. 2022 May 17;17(5):e0265378. doi: 10.1371/journal.pone.0265378 (PMC9113590; doi:10.1371/journal.pone.0265378)
Supplement: Attachment — Submitted filename: Response to reviewers.docx [file pone.0265378.s003.docx]

**Rebuttal letter**

**RESPONSE TO ACADEMIC EDITOR AND REVIEWER**

**Reviewer #2:**

**The methodology is detailed and transparently exposes the design and process followed. In this sense, in the section on methodological rigor, it would be necessary to clarify certain aspects:
1- It is mentioned that maximum heterogeneity was sought in the sample. This is a good thing, since saturation is achieved in a sample with great variability. But it would be necessary to demonstrate this variability. It would be advisable to include a table with characteristics of the sample in which this variability is evidenced.**

**RESPONSE.** In the first version submitted to PLOS ONE, we included a Table 1, which precisely detailed the characteristics of the sample. However, in the Additional Editor Comments, we received the following suggestion: "Table 1: Is potentially quite identifiable. I would delete the same and add a description in the text, ensuring that details cannot identify persons involved in the interview".

For this reason, we then withdrew Table 1, and included the description in the text, as suggested. We leave it to the editors to decide whether or not to publish it. Our opinion is in agreement with what was suggested in Additional Editor Comments, since we have included several tables and figures, and perhaps it would be better to reduce their number as much as possible.

**2- It is reported that the discussion within the team was facilitated at the time of the analysis and mention is made of triangulation. It should briefly detail what type of triangulation and how it was performed.**

**RESPONSE.** We referred to researcher triangulation. As we explained in the manuscript, the principal investigator had extensive knowledge of PHC and this made it convenient for the research team to carry out a frequent exercise of reflection in order to control possible biases. This exercise among the researchers was applied during the coding and categorization process, and also in the interpretation of the results, through a second review by another researcher of the data proposed by the principal investigator, contrasting later those aspects within the team that were doubtful or even divergent.

We appreciate this comment and include this detail in the text:

“researcher triangulation, which was applied during the coding and categorization process, and also in the interpretation of the results, through a second review by another researcher of the data proposed by the main researcher. Any aspects that were doubtful or divergent were further discussed within the team”.

**Reviewer #3:**

**1.- Regarding the choice of this grounded theory design for this study, how do you define and describe your research position in this study?**

**RESPONSE.** The decision to adopt Grounded Theory was made within the research team, after discussion and analysis of other possible options (such as the phenomenological approach and ethnography), which were discarded because the researchers, supported by the expert knowledge of the principal researcher on PHC, realized that in the whole process of configuration of the new role, an explanatory theory of this evolution could be found, and therefore, the decision was made to select the Grounded Theory design.

The position of the principal investigator in this work was determined by her extensive knowledge of primary care, with constant contact over the years with its professionals.

To clarify this point, we include this information in the manuscript, in the section on data analysis, in the following terms:

“which among other aspects, facilitated the decision to opt for Grounded Theory”.

**2.- Could you please explain how you maintain the study rigor? For example, when validating the result to the participants.**

**RESPONSE.** Rigor was generally maintained, respecting the criteria indicated by Strauss & Corbin (2008) and Calderón, C., (2002). Specifically, the validation of the participants' results was carried out by returning them to the informants, that is, the results of the work were communicated to the person who conducted the pilot interview, as well as to the key informant, identifying and confirming both the coherence (credibility) with their own reality.

In the manuscript we have revised and edited the wording to make it clearer, as follows:

“For this purpose, the results were also returned to the informants, that is, the results of the work were communicated to the nurse who conducted the pilot interview, as well as to the key informant, both of whom identified and confirmed the coherence (credibility) with their own reality”.

**3.- The authors mention about the second interview. It is necessary to state whether the interview was undertaken to existing participants or additional ones? please, explain.**

**RESPONSE.** Firstly, in order to achieve an approximation to the studied reality, a first interview or pilot interview was carried out, which was later treated like the rest of the data. For this first interview, a nurse was selected who started in PHC with the new model, by means of an exam and with practically no previous work experience. At the time of the interview, she was still working at the same Health Center. In addition, another nurse interviewed was considered a key informant, as she coincided with a diversity of circumstances that gave her a broad vision in relation to the contributions to the study. Both were participants included in the sample.

**4.- Under the ethical consideration section, I suggest the authors state the number of the letter (of the ethics approval).**

**RESPONSE.** Unfortunately, we are unable to provide a number of the approval letter because at the time the fieldwork was conducted, there was no registration number or code listed on that letter (in Cantabria region).

**5.- During the research study process, two languages (English and Spanish) have been used, thus the authors need to briefly explain the translation process. The readers need to know whether it was undertaken during analytical process or after the study was concluded (when the final theory is emerged).**

**RESPONSE.** Only Spanish was used during the study. The manuscript was then translated into English by a scientific translation service for manuscripts.

Perhaps some of the expressions used may have caused the doubts raised by the reviewer, and therefore we will review the wording.

**6.- Finally, the authors need to highlight the study originality that would help strengthen the paper's conclusions and contribution.**
**RESPONSE.** We appreciate this comment which will certainly strengthen this research. We have added the following paragraph in the Rigor section following this suggestion:

“and, in addition, through two fundamental aspects: first, the application of Bourdieu's theoretical framework to the practice of health care, and second, the results obtained, which illustrate in an operative manner the elements that favor and those that hinder the development of the professional role”.
